# Prevention of Immune Cell Apoptosis as Potential Therapeutic Strategy for Severe Infections

**DOI:** 10.3201/eid1302.060963

**Published:** 2007-02

**Authors:** Janie Parrino, Richard S. Hotchkiss, Mike Bray

**Affiliations:** * National Institutes of Health, Bethesda, Maryland, USA; † Washington University School of Medicine, Saint Louis, Missouri, USA

**Keywords:** Lymphocyte apoptosis, septic shock, therapy, anthrax, plague, Ebola virus, biodefense, synopsis

## Abstract

Some labile cell types whose numbers are normally controlled through programmed cell death are subject to markedly increased destruction during some severe infections. Lymphocytes, in particular, undergo massive and apparently unregulated apoptosis in human patients and laboratory animals with sepsis, potentially playing a major role in the severe immunosuppression that characterizes the terminal phase of fatal illness. Extensive lymphocyte apoptosis has also occurred in humans and animals infected with several exotic agents, including *Bacillus anthracis*, the cause of anthrax; *Yersinia pestis*, the cause of plague; and Ebola virus. Prevention of lymphocyte apoptosis, through either genetic modification of the host or treatment with specific inhibitors, markedly improves survival in murine sepsis models. These findings suggest that interventions aimed at reducing the extent of immune cell apoptosis could improve outcomes for a variety of severe human infections, including those caused by emerging pathogens and bioterrorism agents.

## Abstract 2

Lymphocyte apoptosis prevention may improve survival.

Despite success in controlling many infectious diseases, efforts to defend against the wide range of microbes that threaten human health continue to be challenged by the unexpected emergence of novel pathogens and possible use of a variety of virulent agents as biologic weapons. A defensive strategy based solely on developing new vaccines and antimicrobial and antiviral drugs, each specific for only 1 or a few agents, is unlikely to be successful in dealing with potential microbial threats and will be exceedingly expensive. An alternative approach attempts to identify mechanisms shared by most or all severe infections that could be targets for pharmacologic intervention. Such generic therapies could supplement agent-specific treatment by increasing resistance to infection, potentially improving outcomes for patients in a variety of disease states.

One physiologic process that characterizes several severe infections is a massive loss of lymphocytes, dendritic cells, gastrointestial epithelial cells, and other cell types through apoptosis, or programmed cell death. This process is an apparent acceleration or dysregulation of the same process by which these cell populations are regulated during normal health (*1*,*2*). By impairing the development of adaptive immune responses needed for recovery, the apoptotic destruction of lymphocytes and dendritic cells could have a particularly adverse effect on disease outcome. Fortunately, because programmed cell death is an orderly biochemical process triggered by specific stimuli and executed by a limited range of enzymes, it could be inhibited through pharmacologic countermeasures, offering a novel approach to therapy.

We begin this article by summarizing evidence that a massive apoptotic loss of lymphocytes takes place in humans during the course of septic shock and describing similar findings in animal models of sepsis. Data are then presented that indicate that a marked die-off of lymphocytes also occurs in Ebola hemorrhagic fever, anthrax, and plague, which suggests that unregulated apoptosis of these cells is a component of many, and perhaps all, severe infectious processes and may contribute to high case fatality rates by impairing adaptive immune function. After describing encouraging results obtained in proof-of-concept tests of multiple antiapoptotic interventions in lethal murine models of sepsis, we note some potential limitations of such therapy that could slow its introduction into the therapeutic regimen. Whatever the potential role of such strategies, improved understanding of the causes, time course, and extent of programmed cell death will aid management of patients with severe infections.

## Mechanism and Regulation of Apoptosis

**Figure 1:**
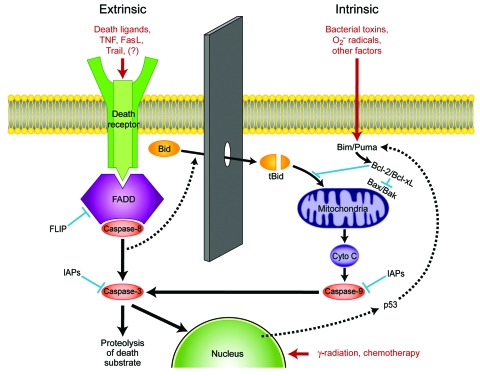
Apoptotic pathways of cell death. The extrinsic pathway is mediated by a variety of death receptor ligands, including tumor necrosis factor (TNF) and Fas ligand (FaSL), that trigger apoptosis by binding to cell surface receptors. In the intrinsic pathway, several adverse factors act upon mitochondria to cause loss of the mitochondrial membrane potential, resulting in leakage into the cytosol of cytochrome C (Cyto C), which together with apoptotic protease activating factor 1 forms the apoptosome that activates caspase-9. Communication between the pathways exists through cleavage of Bcl-2 interacting domain (Bid) by active caspase-8 to form truncated Bid (tBid). Inhibitors of apoptosis (IAPs) can prevent caspase activation under certain conditions. Trail, tumor necrosis factor-α–related apoptosis-inducing ligand; Bim/Puma, Bcl-2 interacting mediator of cell death/p53-upregulated modulator of apoptosis; FADD, Fas-associated death domain; FLIP, Fas-associated death domain-like interleukin-1β converting enzyme-like inhibitory protein.

Apoptosis, or programmed cell death, is the method by which tissue remodeling takes place during normal growth and development and the physiologic mechanism by which labile cell populations such as gastrointestinal epithelial cells, lymphocytes, dendritic cells, and neutrophils are regulated. Apoptosis is of particular importance for the immune system as the means by which self-recognizing lymphocytes are deleted and expanded lymphocyte populations are reduced at the conclusion of an acute immune response (*3*). This closely regulated, energy-requiring process can be initiated through 2 different mechanisms, each based on the successive activation of preexisting but dormant cysteine-aspartate proteases, or caspases (Figure 1).

As its name implies, the intrinsic apoptotic pathway begins within the cell, when toxic alterations bring about a decrease in mitochondrial transmembrane potential, leading to the opening of mitochondrial membrane pores and the release of cytochrome C and other substances into the cytoplasm. The extrinsic pathway, by contrast, is triggered by extracellular events through the binding to cell surface receptors of tumor necrosis factor (TNF) superfamily death ligands, including TNF-α and Fas ligand. Although the intrinsic pathway involves early activation of caspase-9, and the extrinsic pathway is mediated through caspase-8, both lead to activation of the executioner caspase-3 and a variety of proteases and endonucleases. Once begun, apoptosis may be described as an orderly disassembly of the cell from within. Chromosomal DNA is cleaved into oligonucleosomal segments, the nucleus is divided into discrete subunits, and the cell itself is partitioned into multiple membrane-bound fragments whose outer surfaces are marked by large numbers of phosphatidylserine molecules, leading to their rapid uptake by phagocytes. Because all multicellular organisms use programmed cell death to maintain or modify their tissues, this process does not evoke an inflammatory response, and its end products actually serve as antiinflammatory stimuli. Apoptosis thus differs markedly from necrosis, the chaotic breakdown resulting from trauma and other types of damage, in its morphologic and immunologic features (Table 1). Necrosis is characterized by the early loss of outer membrane function, rapid cytoplasmic swelling and disintegration, and release of cell contents into surrounding tissues, which evoke an intense inflammatory response.

**Table 1:** Distinguishing features of apoptosis and necrosis

Feature	Apoptosis	Necrosis
General description	Genetically programmed, orderly process of cell death	Accidental cell death caused by acute injury or other exogenous effect
Membrane integrity	Preserved until late in cell breakdown process	Early loss results in cell and organelle swelling and rupture
Chromosomal DNA	Cleavage at nucleosomes produces ladder pattern on an agarose gel	Random fragmentation produces smear pattern
Inflammatory response	None; products have antiinflammatory effect	Release of intracellular contents causes acute inflammatory response

A large number of cell-surface and cytoplasmic proteins participate in the detection and processing of signals that tip the balance toward or away from programmed cell death. These include members of the Bcl-2 protein family, which have both proapoptotic and antiapoptotic activity (Bcl-2 is antiapoptotic), and other inhibitors (Figure 1). Despite these elaborate control mechanisms, innate or acquired defects in the control of apoptosis may lead to a variety of disease states. For example, excessive inhibition of apoptosis is an underlying mechanism of cancer, while an inappropriate increase is seen in some neurodegenerative diseases and other conditions.

## Lymphocyte Apoptosis in Sepsis

During normal health, the immediate fate of each lymphocyte is determined through continuous summation of a stream of proapoptotic and antiapoptotic signals that arrive from its external environment and from its internal cytoplasmic milieu. A shift toward initiation of apoptosis should therefore be expected during the early phase of sepsis, when bacteria or their byproducts stimulate macrophages to release proapoptotic substances such as TNF-α, nitric oxide, and glucocorticoids. As the disease develops, accumulating products of lymphocyte apoptosis can act as antiinflammatory stimuli, which contribute to the immunosuppression commonly observed as sepsis progresses to septic shock, and which can lead to a state of immune paralysis before death (*2*,*3*).

**Figure 2:**
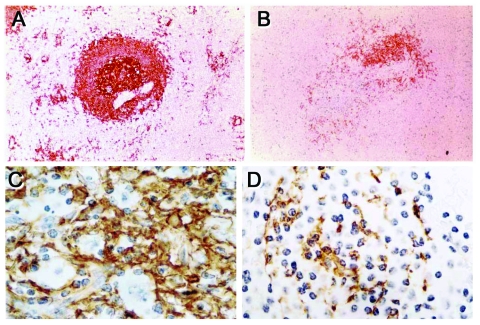
Immunohistochemical identification of B cells and follicular dendritic cells in spleens of patients dying of trauma or sepsis. Total B cells are decreased in the spleen of a patient with sepsis (B) compared with that of a trauma patient (A) (magnification ×400). Similarly, follicular dendritic cells are decreased in the spleen of a patient with sepsis (D) compared with that of a trauma patient (C) (magnification ×600).

Numerous studies have demonstrated a massive apoptotic loss of lymphocytes during sepsis. A prospective investigation in adult patients compared spleens obtained either intraoperatively or within 6 hours after death from sepsis or trauma and found that those from sepsis patients showed a marked decrease in B cells and CD4 T cells (Figure 2) (*1*). The degree of splenic B-cell depletion corresponded with the duration of sepsis. Active caspase-9 was present in splenic lymphocytes with apoptotic features, suggesting a mitochondrial-mediated pathway of cell death, although evidence indicates that apoptotic cell death in patients with sepsis can also proceed by the death receptor pathway (*4*). In most patients, loss of cells from the spleen corresponded with a premortem decrease in circulating lymphocytes.

These findings were closely paralleled in another postmortem study, which showed that B and T cells and dendritic cells were markedly depleted in lymphoid organs of children dying of sepsis and that >3% of cells exhibited histologic signs of apopotosis (*5*). Approximately 15% of patients had prolonged lymphopenia during their terminal course. This report suggested a possible stimulus for apoptosis, in the form of persistent hypoprolactinemia, because prolactin up-regulates expression of the antiapoptotic protein Bcl-2. A third study also noted a profound loss of B and T cells in the spleens of neonates who died of sepsis and chorioamnionitis. Another study compared premortem blood counts in patients with septic shock, sepsis without shock, or nonseptic critical illness and found that increased lymphocyte apoptosis began early in septic shock, and that severe lymphopenia was predictive of a fatal outcome (*6*,*7*).

Extensive loss of lymphocytes through programmed cell death has also been demonstrated in animal models of lethal sepsis induced either by normal intestinal flora or by specific gram-negative bacteria. Studies using cecal ligation and perforation (CLP) in mice have shown profound lymphocyte apoptosis in multiple organs, including the thymus and spleen (*8*). Massive lymphoid apoptosis in the spleen and lymph nodes was also observed in baboons that developed fatal septic shock after injection of *Escherichia coli* (*9*).

## Lymphocyte Apoptosis in Ebola Hemorrhagic Fever

In addition to occurring during common forms of sepsis, a marked increase in lymphocyte apoptosis has been observed in such exotic illnesses as Ebola hemorrhagic fever. When transferred to humans from an unidentified animal reservoir, Ebola virus replicates rapidly in macrophages and dendritic cells, causing intense inflammation, high viremia, and spread of infection to multiple organs, with fever, coagulation abnormalities, and shock (*10*). Case fatality rates have reached 90% in outbreaks in central Africa.

Limited data from patients and more extensive data from laboratory animals indicate that massive lymphocyte apoptosis occurs during Ebola hemorrhagic fever and may contribute to the high death rate. Thus, the few patients who survive infection develop antibodies to the virus during the second week of illness, while fatally infected persons apparently undergo terminal immunosuppression similar to that seen with septic shock (*11*,*12*). A small study of blood samples from patients in Gabon showed that fatal cases of Ebola hemorrhagic fever were characterized by extensive intravascular apoptosis, particularly of T cells, beginning at least 5 days before death, with a decrease and eventual disappearance of Bcl-2 mRNA expression (*11*). In survivors, by contrast, Bcl-2 mRNA was identified in circulating cells during T-cell activation. Importantly, a similar loss of Bcl-2 has been reported in circulating lymphocytes of patients with sepsis (*4*).

Because of the difficulty of performing clinical research under the conditions of an Ebola outbreak, the pathogenesis of lethal infection has been elucidated principally through intensive studies in nonhuman primates, which develop uniformly lethal illness resembling fatal hemorrhagic fever in humans. Lymphocytes in these animals remain free of viral infection but nevertheless undergo extensive apoptosis, with early development of lymphopenia and depletion of circulating natural killer cells and CD4+ and CD8+ lymphocytes (*13*). Massive lymphocyte apoptosis is also observed histologically in lymph nodes, spleen, and other lymphoid tissues, beginning by day 3 postinfection. A model of Ebola virus infection in mice has demonstrated extensive lymphocytolysis in lymph nodes, spleen, and thymus, with histologic features suggestive of apoptosis (*14*). Lymphocyte apoptosis has also been demonstrated in vitro in cultures of Ebola virus–infected peripheral blood mononuclear cells, which suggests that infected monocytes release substances that induce apoptosis in neighboring lymphocytes (*15*).

## Lymphocyte Apoptosis in Anthrax

In inhalational anthrax, spores of *Bacillus anthracis* are carried by pulmonary macrophages to mediastinal lymph nodes, where their replication results in local tissue injury, bacteremia, shock, and death (*16*). The ability of the organism to cause rapidly overwhelming infection suggests that, as in the case of Ebola hemorrhagic fever, immunosuppression plays a role in lethal illness. Few data are available from human cases to assess whether accelerated lymphocyte apoptosis contributes to this process, but a review of autopsy findings from 41 known cases of inhalational anthrax in a 1979 outbreak in Svedlorsk, Russia, showed massive lymphocytolysis in mediastinal lymph nodes and spleens that were morphologically consistent with apoptosis (*17*).

Experimental evidence shows that lethal toxin (LT), an important virulence factor encoded by *B*. *anthracis*, interferes with intracellular signaling and can induce apoptosis. Ultrastructural analysis and terminal deoxynucleotidyl (TUNEL) staining of LT-treated human monocyte–derived dendritic cells found activation of apoptotic pathways (*18*). The same authors demonstrated that bone marrow dendritic cells from C57BL/6 and BALB/c mice differed in susceptibility to LT: cells derived from C57BL/6 mice underwent apoptosis and LT caused necrosis of equivalent cells from BALB/c mice.

## Lymphocyte Apoptosis in Plague

The gram-negative bacillus *Yersinia pestis* causes 2 principal forms of illness in humans, a localized infection of lymph nodes (bubonic plague) and a highly lethal septicemia that is a particularly fulminant form of septic shock (*19*). The striking virulence of *Y*. *pestis* in humans is attributable to a collection of outer membrane proteins (Yops) that cause immune suppression and trigger apoptosis (*20*). Patients dying of plague would therefore be expected to demonstrate increased lymphocyte apoptosis, but data to support this hypothesis are lacking. However, our laboratory studies using a murine model of intranasal *Y*. *pestis* infection have provided evidence of increased lymphocyte apoptosis in the spleen by 36 hours after infection (*21*) (R Hotchkiss, V Miller, unpub. data).

YopH protein inhibits T cell activation by blocking early phosphorylation events necessary for signal transduction through the antigen receptor (*22*). In tests with primary T cells or Jurkat T leukemia cells, the extended presence of YopH led to apoptosis through a mitochondria-dependent pathway, as indicated by mitochondrial breakdown, caspase activation, DNA fragmentation, and annexin V binding. Cell death could be blocked through coexpression of Bcl-x_L_, an antiapoptotic protein in the Bcl-2 family, or by treatment with caspase inhibitors. Evidence of induction of apoptosis was also found in a plague model in rats, in which increased numbers of caspase-positive cells were noted in lymph nodes 36 hours after infection, most prominently in nodes containing the greatest number of bacteria, which suggests Yop-mediated apoptosis (*23*). However, the apoptotic cells could not be identified because of extensive tissue destruction. Multifocal lymphocytolysis was also observed in the white pulp of the spleen, with resultant loss of periarteriolar lymphoid sheath–associated lymphocytes.

## Experimental Inhibition of Apoptosis

Efforts to prevent excessive lymphocyte apoptosis during severe infection have focused either on modification of the signal processing system to create an inherent bias against the triggering of cell death pathways or on inhibition of caspase activity to block their execution. Proof-of-concept experiments with murine sepsis models have shown that both approaches can improve survival. Several studies have shown that transgenic mice overexpressing the antiapoptotic protein Bcl-2 were completely protected against lymphocyte apoptosis in T cells and partially protected in B cells after CLP and showed an increase in survival (*24*,*25*). The exact protective mechanisms, however, are unclear. The authors of 1 report argued that the beneficial effect of Bcl-2 did not depend on prevention of lymphocyte apoptosis because adoptive transfer of myeloid cells overexpressing Bcl-2 also resulted in improved survival after CLP of Rag-1−/− mice, which lack mature T and B cells (*25*). This finding suggests that protection resulted from the release of cytoprotective or antiinflammatory molecules from Bcl-2-overexpressing cells, from an increase in neutrophils at sites of infection, or both. Despite these findings, recent studies that showed a lower death rate after CLP in transgenic mice expressing the antiapoptotic protein Akt in T cells have added further support to the concept that prevention of lymphocyte apoptosis is an independent survival factor in sepsis (*26*).

**Figure 3:**
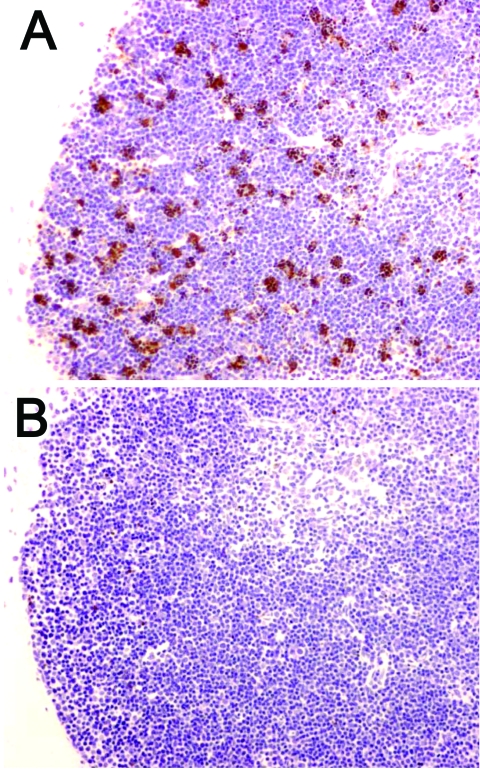
Decreased apoptosis caused by overexpression of Bcl-2 protein in a mouse model of plague. Wild-type mice (A) and mice that overexpressed Bcl-2 in lymphocytes (B) were injected intranasally with *Yersinia pestis*. Thymuses were obtained at 72 h postinfection and stained by using the terminal deoxynucleotidyl method as a marker of apoptotic cell death. Note the decrease in apoptotic cells in the thymus of the Bcl-2 transgenic mouse (magnification ×400).

In addition to these reports that used the CLP model, preliminary studies have shown that Bcl-2 overexpression prevents lymphocyte apoptosis in mice infected with *Y*. *pestis* (R. Hotchkiss, unpub. data). Bcl-2 transgenic mice that overexpressed Bcl-2 in T and B lymphocytes had a marked decrease in splenocyte apoptosis at 72 hours after *Y*. *pestis* infection compared with wild-type animals (Figure 3). These findings provide hope that apoptotic cell death in plague may be preventable by a Bcl-2–based therapy.

Pharmacologic interventions have also been used to prevent initiation of lymphocyte apoptosis in murine models of sepsis (Table 2). One approach has aimed to block initial triggering of the extrinsic pathway by preventing cellular synthesis of Fas or FasL or by administering an inhibitor of Fas-FasL binding. Both techniques have shown benefit in murine CLP studies. Preliminary studies by Chung et al. demonstrated that mice genetically deficient in FasL showed better survival after CLP than their wild-type counterparts (*34*), and a survival benefit was also observed when mice were treated with siRNA to block intracellular synthesis of Fas (*28*). Markedly improved survival was also observed when a Fas receptor fusion protein was injected subcutaneously 12 hours after CLP to act as a decoy for FasL binding. Detailed studies have shown that this intervention restores normal immune function, improves cardiac output, and lowers the serum level of the antiinflammatory cytokine interleukin-10 (*27*).

**Table 2:** Antiapoptotic therapeutic approaches for prevention of lymphocyte apoptosis in murine models of sepsis

Strategy	Intervention	Reference
Prevent triggering of extrinsic pathway	Blockade of Fas ligand by using Fas fusion protein	(*27*)
Prevent triggering of extrinsic pathway	Prevent Fas expression by using siRNA	(*28*)
Prevent initiation	Anti-CD40 agonist antibodies	(*29*)
Prevent initiation	Treatment with Bcl-2 agonist peptides	(*30*)
Prevent triggering of intrinsic pathway	Antiretroviral protease inhibitors	(*31*)
Prevent execution phase	Anticaspase-8 siRNA	(*28*)
Prevent execution phase	Treatment with caspase inhibitors	(*32*,*33*)

Another strategy aims to influence intracellular signaling networks in a direction opposing the initiation of programmed cell death. A recent publication by the Hotckhiss group showed that this could be achieved by exploiting the normal CD40 regulatory pathway through which lymphocytes are stimulated in antiapoptotic directions to produce clonal expansion and functional maturation (*30*). Mice treated with a monoclonal antibody that binds to and stimulates the CD40 receptor showed up-regulation of the antiapoptotic protein Bcl-x_L_, an absence of apoptosis of B cells, a decrease in loss of T cells, and a resistance to CLP (*29*).

Efforts have also been made to alter intracellular signaling by introducing active portions of Bcl-x_L_ fused to carrier peptides to facilitate its transport into cells. In a murine CLP model, treatment resulted in a decrease in lymphocyte apoptosis, but the effect was less marked than that observed in transgenic animals constitutively expressing the same protein (*30*). Another approach has used the licensed HIV protease inhibitors nelfinavir and ritonavir, which in addition to blocking the cleavage of HIV propeptides have direct antiapoptotic effects (*31*). These effects were initially assumed to result from caspase inhibition, but further studies showed that these drugs prevent initiation of the intrinsic apoptotic pathway by stabilizing the mitochondrial membrane potential. Oral administration of nelfinavir and ritonavir to mice, beginning either before or 4 hours after CLP, resulted in decreased lymphocyte apoptosis and improved survival (*31*). Because both drugs are licensed for use in humans, this approach could potentially be evaluated in sepsis patients.

Efforts to block completion of the programmed cell death process by blocking executioner caspases have also been reported. Studies with the broad-spectrum caspase inhibitor zVAD showed decreased apoptosis and improved survival in a mouse CLP model (*32*). Similarly, a selective caspase-3 inhibitor decreased blood bacterial counts and improved survival in mice with sepsis (*33*). Treatment of septic Rag 1−/− mice with caspase inhibitors failed to improve survival, which suggests that the beneficial effect required the presence of lymphocytes.

## Potential Limitations of Antiapoptotic Therapy

Although the proof-of-concept studies described above have shown promising results, deliberate inhibition of apoptosis during severe infections might have unexpected and undesired consequences. One potential adverse effect of antiapoptotic therapy involves its effects on pathogen replication. Some intracellular agents, such as poxviruses, actively inhibit apoptosis of their host cells so as to permit their own continued replication. Theoretically, pharmacologic inhibition of apoptosis in those situations could actually worsen the clinical outcome by providing an advantage to the pathogen. It may therefore be essential to identify the causative agent of infection before initiating antiapoptotic therapy. An alternative approach that may offer several advantages is targeted delivery of antiapoptotic molecules. Similar to current immune-based therapies, apoptosis inhibitors could be directed to specific classes of immune cells, for example by conjugating them to antibodies to CD4 or CD20, thus avoiding adverse consequences (*35*).

Other potential limitations of antiapoptotic therapy relate to possible undesired effects of the use of caspase inhibitors. First, because only a small amount of activated caspase-3 is sufficient to initiate genomic DNA breakdown and lead to apoptotic cell death, a high degree of inhibition would be needed to achieve therapeutic effectiveness (*36*). This requirement presents a therapeutic challenge because of the need for persistent and nearly complete caspase blockade. In addition, there is increasing recognition that caspases have numerous functions in addition to their roles as mediators of programmed cell death. One subset of caspases is critical for regulation of inflammation by processing proinflammatory cytokines such as interleukin-1β; others are essential for lymphocyte activation, proliferation, and protective immunity (*37*,*38*). Patients with defects in caspase-8, for example, are immunodeficient and have recurring infections (*39*). Blocking caspases might therefore have some beneficial effects in decreasing lymphocyte apoptosis in sepsis, but these could be counterbalanced by adverse effects on the ability of the patient to mount an effective immune response. Finally, that inhibition of caspases might induce hyperacute TNF-induced shock in certain situations has been recently reported (*40*). In view of the possible deleterious effects of using caspase inhibitors to treat sepsis, therapy directed at a temporary inhibition of specific caspases, such as caspase-3 or capase-12, timed to either the hyperinflammatory phase or the hypoinflammatory phase of sepsis, might be the most effective approach.

## Conclusions

A massive loss of lymphocytes and other cells through apoptosis is a proven component of the physiologic changes that occur over the course of septic shock. This process appears also to occur in a variety of other severe infections, including anthrax, plague, and Ebola hemorrhagic fever, which are of major concern for biodefense. A variety of proof-of-concept studies with murine sepsis models have demonstrated that this host response worsens disease outcome because its prevention through genetic modification or pharmacologic intervention improves survival. Research is needed to assess the possible contribution of lymphocyte apoptosis to immune impairment in other disease processes, including a variety of newly emerging infections. By helping to bolster immune function, the development of antiapoptotic therapies could mitigate the consequences of infection by a wide variety of pathogenic agents.
